# The complete mitochondrial genome Hainan Gymnure *Neohylomys hainanensis*

**DOI:** 10.12688/f1000research.131600.1

**Published:** 2023-03-16

**Authors:** Feiyun Tu, Xiaodan Hou, Qiujie Lu, Xiaofei Zhai

**Affiliations:** 1 Ministry of Education Key Laboratory for Ecology of Tropical Islands, Key Laboratory of Tropical Animal and Plant Ecology of Hainan Province, College of Life Sciences, Hainan Normal University, Haikou, China

**Keywords:** Neohylomys hainanensis, mitochondrial genome, phylogeny, Erinaceidae

## Abstract

The Hainan Gymnure *Neohylomys hainanensis* is a small-size mammal which occurs in Hainan, China and north Vietnam. Here, we report the complete mitochondrial genome of *N. hainanensis.* The whole mitochondrial genome is 17,337 bp, and contains 13 protein-coding genes, 22 tRNAs, 2 rRNAs and one control region. The base composition of the *N. hainanensis* total mitogenome is: 33.4% A, 12.2% G, 33.1% T, and C 21.3%, with an A + T content of 66.5%. The K2P genetic distance analysis supports current taxonomy that places the *hainanensis*, *sinensis* and *suillus* in different genera. Phylogenetic analysis suggests that *N* *. hainanensis* is closely related to *Neotetracus sinensis* based on the complete mitochondrial genome sequences. The mitogenomic data will contribute to molecular phylogenetics and conservation genetics of the species.

## Introduction

The Hainan Gymnure *Neohylomys hainanensis* Shaw and Wang, 1959 is mainly distributed in Hainan, China and north Vietnam ( Liu and Wu 2019; Abramov *et al*. 2018). It mainly inhabits the tropical forests and subtropical forests ( Smith *et al*. 2009). To date, the phylogenetic relationships of three species ( *N. hainanensis*, *Hylomys suillus* and *Neotetracus sinensis*) within Erinaceidae have been debated ( Cobert 1988; Gould 1995; Grenyer and Purvis 2003; He *et al*. 2012). Previous studies ( Cobert 1988; Gould 1995; Grenyer and Purvis 2003) indicated sister relationships of *N. hainanensis* and *N. sinensis* based on morphological data. However, He *et al*. (2012) showed that *N. sinensis* clustered with *H. suillus* rather than *N. hainanensis* based on combined data of mitochondrial genes ( *CYTB*, *ND2* and *12S*). Complete mitochondrial genome sequences are highly informative markers for resolving high level phylogenetic relationships among taxa ( Kumazawa 2007; Shen *et al*. 2010; Yue *et al*. 2015). Though the complete mitochondrial genome of *N. hainanensis* is available in GenBank (MW429379), we sequenced the mitochondrial genome using our sample based on primer walking method to confirm the accuracy of the data. Additionally, we investigated phylogenetic relationships of *N. hainanensis* among family Erinaceidae to solve the disputes.

## Methods

The animal was captured using a mouse cage trap at Yinggeling Mountains (109.289°E, 18.878°N), Hainan province, China in May, 2022. The animal was euthanized in the euthanasia cage (10L), which was gradually filled with 99% purity of CO _2_ within 2-3 minutes.

A total of 2 g of femoral muscle sample was clipped from the specimen using surgical scissors, and the total genomic DNA was extracted from the muscle tissue using the phenol–chloroform extraction (Sambrook *et al*. 1989), which contained three main procedures: 1) Sodium dodecylsulfate (SDS) and proteinase K were used for the enzymatic digestion of proteins; 2) A mixture of phenol: chloroform:isoamyl alcohol (25:24:1) was then added to promote the partitioning of lipids into the organic phase; 3) The DNA was rinsed using analytical alcohol of different solubility and then otained the purified DNA for PCR. Seven primer combinations were used for the generation of PCR products (see *Extended data* ( Tu 2023)). PCR amplifications of mitochondrial genome of *N. hainanensis* were listed as follows: PCR amplifications were carried out in 25 uL reaction volumes containing 1× ^EX^ Taq buffer (Mg ^2+^ Free; TaKaRaBiotech, Dalian, China), 2.5 mM MgCl _2_, 0.1-0.4 mM dNTP, 0.4 uM each primer, 0.04 u/uL ^EX^ Taq polymerase, and ~5 ng/uL total genomic DNA as template. PCR protocol was set as follows: an initial denaturation at 94°C for 4 min; 35 cycles of amplification as the main procedure: a denaturation at 94°C for 45 s, an annealing at 50-60°C for 50 s, an elongation at 72°C for 2-3 min, and a final extension at 72°C for 4 min. All PCR products were examined through electrophoresing on a 1.0% agarose gel and then directly sequenced using an ABI 3730xl sequencer. All sequences were assembled and edited using SeqMan (DNASTAR 7.1.0) (Swindell and Plasterer 1997). The boundaries of protein-coding genes were predicted via comparison with homologs using MEGA 6.0 ( Tamura *et al*. 2013). The transfer RNA (tRNA) genes were identified using tRNAscan-SE 2.0 ( Lowe and Chan 2016).

To better understand the phylogentic position of *N. hainanensis* within Eulipotyphla, we constructed a phylogenetic tree by using neighbor-joining (NJ) method implemented in MEGA6.0 ( Tamura *et al*. 2013) based on 13 complete mitochondrial genome sequences of Eulipotyphla using Kimura 2-parameters (K2P) model.

## Results

The whole mitochondrial genome of *N. hainanensis* is 17,337 bp in length (GenBank Accession number ON054206.2 ( Tu and Hou 2022)) and contains 13 protein-coding genes (PCGs), 22 tRNAs genes, two rRNAs genes, and one control region. The base composition of *N. hainanensis* mitogenome is as follows: A 33.4%, G 12.2%, T 33.1%, and C 21.3%. Typically, an A+T rich pattern (66.5%) was observed. Comparison with a previously published mitochondrial genome sequence (MW429379) for the same species, these two sequences differ in length (17,337 bp vs 16,795 bp), which may be due to mitochondrial control region sequence variations (1,868 bp vs 1334bp). Within 37 mitochodrial genes, the *ND6* gene and eight tRNAs ( *tRNA ^Gln^*, *tRNA ^Ala^*, *tRNA ^Asn^*, *tRNA ^Cys^*, *tRNA ^Tyr^*, *tRNA ^Ser^*, *tRNA ^Glu^* and *tRNA ^Pro^*) were encoded on the L-strand, whereas all other genes were encoded on the H-strand. Most mitochondrial PCGs, use ATG as its start codon, *ND2* and *ND3* begin with ATT, and *ND5* begin with ATA. Five genes ( *COX2*, *ATP6*, *ND3*, *ND4L* and *ND5*) use TAA as stop codons, whereas four genes ( *ND1*, *ND2*, *COX1* and *ATP8*) end with TAG, and *ND6* ends with AGA. *COX3*, *ND4* and *CYTB* use T as an incomplete stop codon.

**Figure 1.**
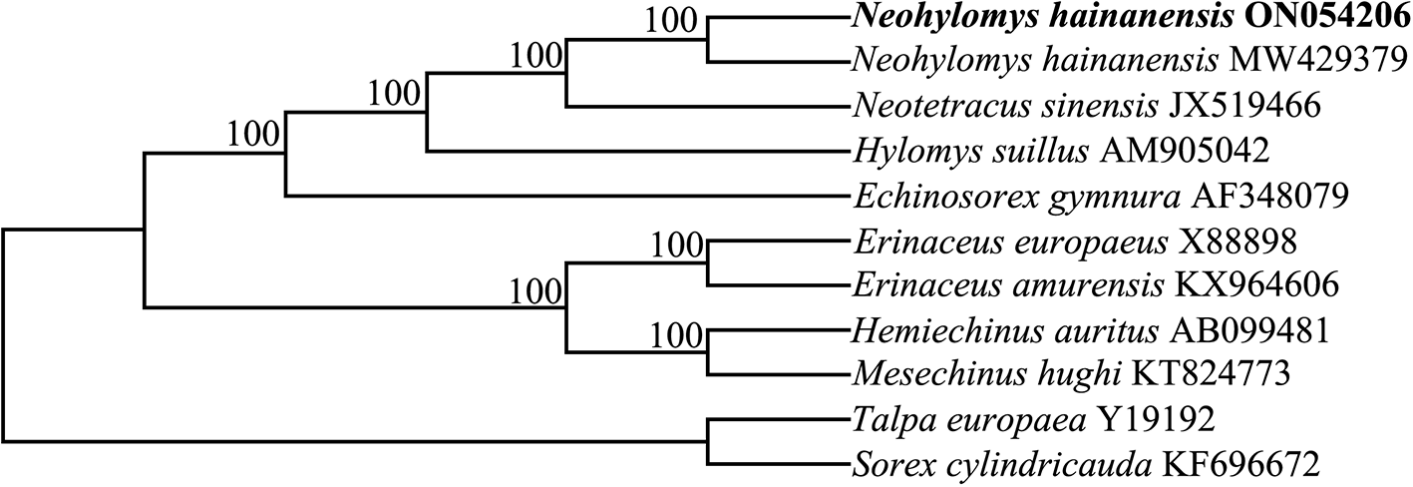
A phylogenetic tree of ten species based on the complete mitochondrial genomes. Nodal support indicated by bootstrap.

A phylogenetic tree showed that two sequences of *N. hainanensis* shared a common evolutionary ancestry. Within subfamily Galericinae, *H. suillus* and the common ancestors of *N. hainanensis* and *N. sinensis* shaped sister groups ( Figure 1). The results were consistent with morphological studies ( Cobert 1988; Gould 1995; Grenyer and Purvis 2003). Our results support current taxonomy that places the *hainanensis*, *sinensis* and *suillus* in different genera ( Smith *et al*. 2009; Wei *et al*. 2021). This mitochondrial genome sequence determined in this study will benefit conservation genetics and phylogenetics of the species in future.

### Ethical statement

This work complies with the International Union for Conservation of Nature (IUCN) policies research involving species at risk of extinction (see Guidelines for appropriate uses of IUCN Red list data version 4.0; https://www.iucnredlist.org/resources/guidelines-for-appropriate-uses-of-red-list-data), as the species under study is an endangered species. All procedures were approved by Animal Research Ethics Committee of Hainan Provincial Education Center for Ecology and Environment, Hainan Normal University (HNECEE-2022-003) on March 6, 2022.

## Data Availability

NCBI Nucleotide: *Neohylomys hainanensis* voucher HN20N051 mitochondrion, complete genome. Accession number ON054206.2, https://www.ncbi.nlm.nih.gov/nuccore/ON054206.2 ( Tu and Hou 2022). Figshare: *Neohylomys hainanensis* primer combinations data. https://doi.org/10.6084/m9.figshare.22180219.v1 ( Tu 2023). This project contains the following extended data:
- Primer information Data are available under the terms of the Creative Commons Attribution 4.0 International license (CC-BY 4.0).
