# Supplementary material for: The complete mitochondrial genome Hainan Gymnure,  Neohylomys hainanensis
Source: F1000Res. 2025 Oct 20;12:294. Originally published 2023 Mar 16. [Version 2] doi: 10.12688/f1000research.131600.2 (PMC12605588; doi:10.12688/f1000research.131600.2)
Supplement: Peer review history

# Reviewer report: Reviewer response for version 2

**DOI:** 10.5256/f1000research.189705.r425492

**Published:** 2026-01-02

**Authors:** Michael Allen

**Affiliations:** 1 Map of Life Solutions, New Brunswick, USA

**GENERAL COMMENTS**

 The manuscript is much improved. My two major concerns are as follows. 1) Choosing a more robust method of tree building (i.e., than the neighbor joining method) would yield a more convincing discussion of evolutionary relationships in the group. Consider adopting a ML or Bayes-based tree building approach. 2) Searching for the species in GenBank yielded 2 genomes for the *Neohylomys hainanensis* voucher HN20N051, only one of which appears to be the subject of this paper. This paper would be stronger if the existence of this second genome for voucher HN20N051 was discussed, acknowledged, and/or explained. 

**DETAILED COMMENTS**

**INTRODUCTION**

**Text:**  “...we constructed a phylogenetic tree by using neighbor-joining (NJ) method implemented in MEGA6.0 (Tamura et al. 2013) based on 13 complete mitochondrial genome sequences of Eulipotyphla using Kimura 2-parameters (K2P) model…”:

**Comment: **While Neighbor-Joining trees are acceptable for exploratory analyses, most published phylogenetic trees based on sequences are built using include Maximum Likelihood (e.g., RAxML) or Bayesian (e.g., BEAST) methods, which are considered more rigorous.

**RESULTS**

**Text:** “Our results support current taxonomy that places the *N. hainanensis*, *N. sinensis* and *H. suillus* in different genera”:

**Comment: **Again, this would be better supported if at least one other methods (ML/Bayes) was implemented in parallel or instead of the NJ method.

**Comment: **It would help readers understand the significance of this contribution to explain why there are 2 mt genomes of *Neohylomys hainanensis* in GenBank, both of the same voucher specimen, and submitted about a year apart. If they are two separate efforts, it would be helpful to discuss how and why they are different. Only one appears to have resulted from this current study. See below for the information yielded by the GenBank search:

 1. *Neohylomys hainanensis* voucher HN20N051 mitochondrion, complete genome

 17,443 bp circular DNA

 Accession: NC_063830.1 GI: 2248514867

 2. *Neohylomys hainanensis* voucher HN20N051 mitochondrion, complete genome

 17,337 bp circular DNA

 Accession: ON054206.2 GI: 2326472219

Are the datasets clearly presented in a usable and accessible format, and the assembly and annotation available in an appropriate subject-specific repository?

Yes

Are sufficient details of the sequencing and extraction, software used, and materials provided to allow replication by others?

Partly

Are the rationale for sequencing the genome and the species significance clearly described?

Yes

Are the protocols appropriate and is the work technically sound?

Yes

Reviewer Expertise:

Conservation, Environmental DNA, DNA metabarcoding, Bioinformatics

I confirm that I have read this submission and believe that I have an appropriate level of expertise to confirm that it is of an acceptable scientific standard.

# Reviewer report: Reviewer response for version 2

**DOI:** 10.5256/f1000research.189705.r427935

**Published:** 2025-11-11

**Authors:** Kai He

**Affiliations:** 1 Guangzhou University, Guangzhou, China

This manuscript reports the complete mitochondrial genome of the Hainan Gymnure, *Neohylomys hainanensis*, a poorly understood galericine mammal.

 The primary and most significant contribution of this work is the generation of a high-quality, complete mitogenome (17,337 bp) using a meticulous primer-walking and Sanger sequencing approach. This method is particularly noteworthy because the authors' resulting sequence is substantially longer than the existing public sequence (MW429379; 16,795 bp).

 As the authors note, this discrepancy is concentrated in the control region. This strongly suggests that the previous sequence, which was generated via NGS, was likely an incomplete assembly that collapsed this complex, repetitive region. Therefore, this paper does not just provide a *second* sequence, but rather the first truly complete and accurately assembled reference mitogenome for this species. For a little-known mammal like *N. hainanensis*, this is a valuable and important genomic resource.

Are the datasets clearly presented in a usable and accessible format, and the assembly and annotation available in an appropriate subject-specific repository?

Yes

Are sufficient details of the sequencing and extraction, software used, and materials provided to allow replication by others?

Yes

Are the rationale for sequencing the genome and the species significance clearly described?

Yes

Are the protocols appropriate and is the work technically sound?

Yes

Reviewer Expertise:

Mammalogy, taxonomy and phylogeny

I confirm that I have read this submission and believe that I have an appropriate level of expertise to confirm that it is of an acceptable scientific standard.

# Reviewer report: Reviewer response for version 2

**DOI:** 10.5256/f1000research.189705.r425493

**Published:** 2025-11-08

**Authors:** Perinçek Seçkinozan Şeker

**Affiliations:** 1 Artvin Çoruh University, Artvin, Turkey

I have carefully reviewed the revised version of the manuscript. My observations regarding several significant omissions are outlined below. 

 ''The authors have partially implemented the edits I suggested in the first revision of the article. However, the justification for the study’s purpose remains insufficient. While the introduction mentions that reporting a new genome sequence would help resolve phylogenetic relationships, this alone does not fully support the significance of the work. It should also be emphasized that the new genome sequence will contribute to understanding the origin and evolution of global biodiversity at the level of genetic diversity specific to the species studied.

 In addition, it would be beneficial to include the genetic distances between the taxa used in the phylogenetic analyses, calculated using the Kimura 2-parameter model. Presenting these values would more clearly highlight the differences between the newly sequenced genome and the existing genome sequence of the same species currently available in NCBI.

 Finally, the concluding sentence should be expanded to specify which deficiencies are being addressed and how the study contributes to overcoming them''.

Are the datasets clearly presented in a usable and accessible format, and the assembly and annotation available in an appropriate subject-specific repository?

Yes

Are sufficient details of the sequencing and extraction, software used, and materials provided to allow replication by others?

Yes

Are the rationale for sequencing the genome and the species significance clearly described?

Partly

Are the protocols appropriate and is the work technically sound?

Yes

Reviewer Expertise:

Molecular Systematics Zoology

I confirm that I have read this submission and believe that I have an appropriate level of expertise to confirm that it is of an acceptable scientific standard.

# Reviewer report: Reviewer response for version 1

**DOI:** 10.5256/f1000research.144457.r250944

**Published:** 2024-05-11

**Authors:** Perinçek Seçkinozan Şeker

**Affiliations:** 1 Artvin Çoruh University, Artvin, Turkey

The authors report the complete mitochondrial DNA sequence of an insectivorous mammal species whose complete mitochondrial genome is already available at NCBI to confirm the accuracy of the data https://www.ncbi.nlm.nih.gov/nuccore/MW429379. At this point, the logic of sequencing the genome of this species and the importance of the species, which are the main issues that will emphasize the importance of the article, have not been sufficiently justified. Although this is even a genome announcement, this section should be developed by clearly stating the rationales. The methods used are up-to-date and fit for the study. The obtained genome sequence has been presented in a usable and accessible format in NCBI. A previously reported genome sequence of this species is available at NCBI. Therefore, it would be better to give the results of statistical analyses (for example, the topology of the phylogenetic tree and K2P distances) and the structures of the genomes comparatively. For example, there is a difference in length between the two genomes Why does this situation occur? It should be presented by comparing. As a result, this study presented as a genome note can be indexed if these requirements are improved.

Are the datasets clearly presented in a usable and accessible format, and the assembly and annotation available in an appropriate subject-specific repository?

Yes

Are sufficient details of the sequencing and extraction, software used, and materials provided to allow replication by others?

Yes

Are the rationale for sequencing the genome and the species significance clearly described?

Partly

Are the protocols appropriate and is the work technically sound?

Yes

Reviewer Expertise:

Molecular Systematics Zoology

I confirm that I have read this submission and believe that I have an appropriate level of expertise to confirm that it is of an acceptable scientific standard, however I have significant reservations, as outlined above.

# Response

**Published:** 2025-10-14

**Authors:** Feiyun Tu

**Affiliations:** Hainan Normal University, China

Thank you for your suggestions.

 The authors report the complete mitochondrial DNA sequence of an insectivorous mammal species whose complete mitochondrial genome is already available at NCBI to confirm the accuracy of the data https://www.ncbi.nlm.nih.gov/nuccore/MW429379. At this point, the logic of sequencing the genome of this species and the importance of the species, which are the main issues that will emphasize the importance of the article, have not been sufficiently justified. Although this is even a genome announcement, this section should be developed by clearly stating the rationales. The methods used are up-to-date and fit for the study. The obtained genome sequence has been presented in a usable and accessible format in NCBI. A previously reported genome sequence of this species is available at NCBI. Therefore, it would be better to give the results of statistical analyses (for example, the topology of the phylogenetic tree and K2P distances) and the structures of the genomes comparatively. For example, there is a difference in length between the two genomes Why does this situation occur? It should be presented by comparing. As a result, this study presented as a genome note can be indexed if these requirements are improved.

 Respond: We have revised the manuscript. We have compared the complete mitochondrial genome of Neohylomys hainanensis and previous reported genome sequence. 

 Comparison with a previously published mitochondrial genome sequence (MW429379) for the same species, these two sequences differ in length (17,337 bp vs 16,795 bp), which may be due to mitochondrial control region sequence variations (1,868 bp vs 1,334bp).

# Reviewer report: Reviewer response for version 1

**DOI:** 10.5256/f1000research.144457.r255823

**Published:** 2024-04-04

**Authors:** Michael Allen

**Affiliations:** 1 Map of Life Solutions, New Brunswick, USA

General comments

 This is a useful study in that it provides new genetic information for a poorly studied endangered species, the mammal *Neohylomys hainanensis*. The mitochondrial genome described therein will prove useful for future phylogenetic research as well as for metabarcoding studies of distribution and habitat use. While there was already a single complete genome for the species available in GenBank, the new genome and additional analysis provided by the authors provides novel information regarding intra- and inter-specific variation within the species and clade. The value of the paper could be enhanced to the extent that the authors can expand and elaborate on these analyses.

 Detailed comments

 TITLE

 Suggest changing the title to the following to improve clarity: “The complete mitochondrial genome of the Hainan Gymnure, *Neohylomys hainanensis*”

 ABSTRACT

 “…the *hainanensis*, *sinensis* and *suillus*…”: These three species should be identified by their full binomial name here, not just the specific epithet. E.g., “ *N. hainanensis, Hylomys suillus and Neotetracus sinensis*”

 It would be good to report more detail here about what type of phylogenetic analysis was performed, if possible.

 INTRODUCTION

 Paragraph 1, sentences 1 & 2: Suggest rephrasing as follows: “The Hainan Gymnure ( *Neohylomys hainanensis* Shaw and Wang, 1959) is a rodent distributed in Hainan, China and north Vietnam, where it inhabits tropical and subtropical forests (Smith et al. 2009; Liu and Wu 2019; Abramov et al. 2018).” I believe this would improve the flow and would serve to inform the reader what type of organism it is (i.e., a Mammal and a Rodent), information which is not currently provided.

 Please state how many species are currently recognized within the family Erinaceidae.

 Paragraph 1, sentence 6: suggest deleting the words “high level” as their meaning is unclear here.

 Paragraph 1, sentence 8: suggest changing “…to solve the disputes.” to “…in an attempt to resolve the current phylogenetic disputes.”

 METHODS

 Methods paragraph 1, sentence 2: I recommend changing “…in the euthanasia cage (10L)…” to “…in a 10 L euthanasia cage…”. And, if possible, describe the cage and its material as the phrase “euthanasia cage” is not self-evident.

 Methods paragraph 2, sentence 1: delete the word “the” from the phrase “using the phenol–chloroform extraction”.

 Methods paragraph 2, “All sequences were assembled and edited using SeqMan (DNASTAR 7.1.0) (Swindell and Plasterer 1997).”: Surely there must be more methods to report here in addition to which software you used. Are there any more details about settings used, etc., you can provide that would help someone better understand and repeat this analysis?

 Methods paragraph 3: “MEGA6.0” should have a space in it.

 Methods paragraph 3 (“13 complete mitochondrial genome sequences of Eulipotyphla”): Please either state which species genomes were used here, or else reference Table 1 which I think provides that information. Also, please address why are there only 11 genomes displayed in Table 1 given that you analyzed 13. It would also be good to report how many species and genera are currently known to occur within Eulipotyphla. That would provide readers a better understanding of how complete your analysis is.

 RESULTS

 Results paragraph 1, sentence 4: sentence is incomplete as written. Please change to “Comparison with a previously published mitochondrial genome sequence (MW429379) for the same species revealed that these two sequences differ in length (17,337 bp vs 16,795 bp), which may be due to mitochondrial control region sequence variations (1,868 bp vs 1334bp).”

 Results paragraph 1, penultimate sentence: Contains minor punctuation and wording issues. Rephrase as follows: “Most mitochondrial PCGs contain ATG as the start codon, ND2 and ND3 begin with ATT, and ND5 begins with ATA.”

 Results paragraph 2, sentence 2: The word “shaped” should be replaced with the word “formed” to improve clarity.

 Results paragraph 2, sentence 4: Please include the genus names or initials for these specific epithets. (See comment in Abstract.)

 Results paragraph 2, last sentence: Change ending of sentence as follows to improve clarity: “…will benefit future research into conservation genetics and phylogenetics for the species.”

 It would be useful to include summary statistics regarding pairwise K2P differences for different regions (e.g., in the context of DNA barcoding and metabarcoding).

 DISCUSSION

 Consider including a brief Discussion section if permitted by the journal format.

 FIGURES

 Figure 1: Please include in the caption the fact that this represents 10 species from the Eulipotyphla clade. Also, briefly explain that they were created using a neighbor-joining tree and that the codes after the species names represent GenBank accession numbers (if true).

 TABLES

 While not required, a table comparing the various attributes of the 2 known genomes of *N. hainanensis* (and perhaps other species analyzed) would be welcome.

Are the datasets clearly presented in a usable and accessible format, and the assembly and annotation available in an appropriate subject-specific repository?

Yes

Are sufficient details of the sequencing and extraction, software used, and materials provided to allow replication by others?

Partly

Are the rationale for sequencing the genome and the species significance clearly described?

Yes

Are the protocols appropriate and is the work technically sound?

Yes

Reviewer Expertise:

Conservation, Environmental DNA, DNA metabarcoding, Bioinformatics

I confirm that I have read this submission and believe that I have an appropriate level of expertise to confirm that it is of an acceptable scientific standard, however I have significant reservations, as outlined above.

# Response

**Published:** 2025-10-14

**Authors:** Feiyun Tu

**Affiliations:** Hainan Normal University, China

TITLE

 Suggest changing the title to the following to improve clarity: “The complete mitochondrial genome of the Hainan Gymnure, *Neohylomys hainanensis* ”

 Respond: We would like to change the title as: The complete mitochondrial genome of the Hainan Gymnure, *Neohylomys hainanensis*

 ABSTRACT

 “…the *hainanensis* , *sinensis* and *suillus* …”: These three species should be identified by their full binomial name here, not just the specific epithet. E.g., “ *N. hainanensis, Hylomys suillus and Neotetracus sinensis* ”

 It would be good to report more detail here about what type of phylogenetic analysis was performed, if possible.

 Respond: We would like to provided full binomial name in the abstract.

 The Hainan Gymnure *Neohylomys hainanensis* is a small-size mammal which occurs in Hainan, China and north Vietnam. Here, we report the complete mitochondrial genome of *N. hainanensis* . The whole mitochondrial genome is 17,337 bp, and contains 13 protein-coding genes, 22 tRNAs, 2 rRNAs and one control region. The base composition of the *N. hainanensis* total mitogenome is: 33.4% A, 12.2% G, 33.1% T, and C 21.3%, with an A + T content of 66.5%. The K2P genetic distance analysis supports current taxonomy that places *N.* *hainanensis* , *Hylomys suillus* and *Neotetracus sinensis* in different genera. Phylogenetic analysis suggests that *N. hainanensis* is closely related to *N* *.* *sinensis* based on the complete mitochondrial genome sequences. The mitogenomic data will contribute to molecular phylogenetics and conservation genetics of the species.

 INTRODUCTION

 Paragraph 1, sentences 1 & 2: Suggest rephrasing as follows: “The Hainan Gymnure ( *Neohylomys hainanensis* Shaw and Wang, 1959) is a rodent distributed in Hainan, China and north Vietnam, where it inhabits tropical and subtropical forests (Smith et al. 2009; Liu and Wu 2019; Abramov et al. 2018).” I believe this would improve the flow and would serve to inform the reader what type of organism it is (i.e., a Mammal and a Rodent), information which is not currently provided.

 Respond: I agree with the revision.

 Please state how many species are currently recognized within the family Erinaceidae.

 Respond: In China, nine species are currently recognized within the family Erinaceidae.

 Paragraph 1, sentence 6: suggest deleting the words “high level” as their meaning is unclear here.

 Respond: We would like to delete the words “high level” in the revised manuscript.

 Paragraph 1, sentence 8: suggest changing “…to solve the disputes.” to “…in an attempt to resolve the current phylogenetic disputes.”

 Respond: I agree with the revision.

 METHODS

 Methods paragraph 1, sentence 2: I recommend changing “…in the euthanasia cage (10L)…” to “…in a 10 L euthanasia cage…”. And, if possible, describe the cage and its material as the phrase “euthanasia cage” is not self-evident.

 Respond: I agree with the revision. Please change “…in the euthanasia cage (10L)…” into “…in a 10 L euthanasia cage…”.

 Methods paragraph 2, sentence 1: delete the word “the” from the phrase “using the phenol–chloroform extraction”. 

 Respond: Thank you for your suggestions. I agree with the revision.

 A muscle sample was obtained from the preserved specimen, and the total genomic DNA was extracted from the muscle tissue using a standard phenol-chloroform extraction protocol (Sambrook et al. 1989).

 Methods paragraph 2, “All sequences were assembled and edited using SeqMan (DNASTAR 7.1.0) (Swindell and Plasterer 1997).”: Surely there must be more methods to report here in addition to which software you used. Are there any more details about settings used, etc., you can provide that would help someone better understand and repeat this analysis?

 Respond: We have changed as: “The complete mitochondrial genome sequence was then assembled and manually refined utilizing SeqMan (DNASTAR 7.1.0) (Swindell and Plasterer 1997) following analysis of chromatogram files”.

 Methods paragraph 3: “MEGA6.0” should have a space in it.

 Respond: Thank you for your suggestions. I agree with the revision.

  To better understand the phylogentic position of *N. hainanensis* within Eulipotyphla, we constructed a phylogenetic tree by using neighbor-joining (NJ) method implemented in MEGA 6.0 ( Tamura et al. 2013) based on 13 complete mitochondrial genome sequences of Eulipotyphla using Kimura 2-parameters (K2P) model.

 Methods paragraph 3 (“13 complete mitochondrial genome sequences of Eulipotyphla”): Please either state which species genomes were used here, or else reference Table 1 which I think provides that information. Also, please address why are there only 11 genomes displayed in Table 1 given that you analyzed 13. It would also be good to report how many species and genera are currently known to occur within Eulipotyphla. That would provide readers a better understanding of how complete your analysis is.

 Respond: We have provided Table 1.

 RESULTS

 Results paragraph 1, sentence 4: sentence is incomplete as written. Please change to “Comparison with a previously published mitochondrial genome sequence (MW429379) for the same species revealed that these two sequences differ in length (17,337 bp vs 16,795 bp), which may be due to mitochondrial control region sequence variations (1,868 bp vs 1334bp).”

 Respond: Thank you for your suggestions. I agree with the revision.

 Results paragraph 1, penultimate sentence: Contains minor punctuation and wording issues. Rephrase as follows: “Most mitochondrial PCGs contain ATG as the start codon, ND2 and ND3 begin with ATT, and ND5 begins with ATA.”

 Thank you for your suggestions. I agree with the revision.

 Results paragraph 2, sentence 2: The word “shaped” should be replaced with the word “formed” to improve clarity.

 Thank you for your suggestions. I agree with the revision.

 Results paragraph 2, sentence 4: Please include the genus names or initials for these specific epithets. (See comment in Abstract.)

 Thank you for your suggestions. I agree with the revision.

 Results paragraph 2, last sentence: Change ending of sentence as follows to improve clarity: “…will benefit future research into conservation genetics and phylogenetics for the species.”

 Respond: Thank you for your suggestions. I agree with the revision.

 It would be useful to include summary statistics regarding pairwise K2P differences for different regions (e.g., in the context of DNA barcoding and metabarcoding).

 Respond: Thank you for your suggestions.

 DISCUSSION

 Consider including a brief Discussion section if permitted by the journal format.

 Respond: Discussion section is not permitted by the journal.

 FIGURES

 Figure 1: Please include in the caption the fact that this represents 10 species from the Eulipotyphla clade. Also, briefly explain that they were created using a neighbor-joining tree and that the codes after the species names represent GenBank accession numbers (if true).

 Respond: Thank you for your suggestions.

 Figure 1. A phylogenetic tree of ten species based on the complete mitochondrial genomes using NJ method. Nodal support indicated by bootstrap. The codes after the species names represent GenBank accession numbers.

 TABLES

 While not required, a table comparing the various attributes of the 2 known genomes of *N. hainanensis* (and perhaps other species analyzed) would be welcome.

 Respond: Thank you for your suggestions.We have provided Table 1.
